# Interaction Analysis of Commercial Graphene Oxide Nanoparticles with Unicellular Systems and Biomolecules

**DOI:** 10.3390/ijms21010205

**Published:** 2019-12-27

**Authors:** Brixhilda Domi, Carlos Rumbo, Javier García-Tojal, Livia Elena Sima, Gabriela Negroiu, Juan Antonio Tamayo-Ramos

**Affiliations:** 1 International Research Centre in Critical Raw Materials-ICCRAM, University of Burgos, Plaza Misael Bañuelos s/n, 09001 Burgos, Spain; bdomi@ubu.es (B.D.); crumbo@ubu.es (C.R.); 2 Department of Chemistry, University of Burgos, Plaza Misael Bañuelos s/n., 09001 Burgos, Spain; qipgatoj@ubu.es; 3 Department of Molecular Cell Biology, Institute of Biochemistry of the Romanian Academy, 060031 Bucharest, Romania; livia_e_sima@yahoo.com (L.E.S.); gabrielanegroiu@yahoo.com (G.N.)

**Keywords:** graphene, unicellular organisms, toxicity, binding capacity, ATR-FTIR, TEM, ICP-MS

## Abstract

The ability of commercial monolayer graphene oxide (GO) and graphene oxide nanocolloids (GOC) to interact with different unicellular systems and biomolecules was studied by analyzing the response of human alveolar carcinoma epithelial cells, the yeast *Saccharomyces cerevisiae* and the bacteria *Vibrio fischeri* to the presence of different nanoparticle concentrations, and by studying the binding affinity of different microbial enzymes, like the α-l-rhamnosidase enzyme RhaB1 from the bacteria *Lactobacillus plantarum* and the AbG β-d-glucosidase from *Agrobacterium* sp. (strain ATCC 21400). An analysis of cytotoxicity on human epithelial cell line A549, *S. cerevisiae* (colony forming units, ROS induction, genotoxicity) and *V. fischeri* (luminescence inhibition) cells determined the potential of both nanoparticle types to damage the selected unicellular systems. Also, the protein binding affinity of the graphene derivatives at different oxidation levels was analyzed. The reported results highlight the variability that can exist in terms of toxicological potential and binding affinity depending on the target organism or protein and the selected nanomaterial.

## 1. Introduction

The interest in the immobilization of microorganisms and microbial enzymes for biotechnological applications has been continuously rising during the last decades because of several factors, including the increased availability of microbial strains and biocatalysts tailored to new applications, the development of new immobilization supports with improved properties, and the need of a shift toward the use of more sustainable processes in different industrial fields [1,2,3,4,5]. The immobilization of microorganisms and enzymes on solid carriers leads to a number of benefits. Immobilized biocatalysts facilitate the efficient recovery and separation of the reaction product, the reutilization of the biocatalyst, and enhance the safety of the material handling (i.e., preventing the appearance of allergies). The use of solid supports of microbial cells for the production of high-value compounds (chemicals, enzymes, etc.) and transformation processes in multiple fields (e.g., agricultural, environmental, food, medical, etc.) has been explored as well to enhance the microbial biological activity, to facilitate their delivery and to separate them more easily from the fermentation broth [3,5,6,7,8]. Therefore, during the last years there has been an emerging interest in biocompatibility studies for interfacing biological systems with artificial materials. Unicellular microorganisms, such as bacteria, fungi, and algae, have been utilized extensively for the encapsulation of whole single cells as well as for the introduction of nanomaterials onto the living cells.

During the last 40 years, a range of different materials have been investigated as enzyme and microbial immobilization matrices: from organic compounds, like natural alginate or carrageenan or synthetic polymers, to inorganic compounds, such as processed or natural minerals, like silica [3,9]. In the last decade, the focus has been put in the use of nanocomposites as promising immobilization matrices. This is, in part, due to the enormous functional surface area they provide, which increases the microbial and enzyme loading. Metal and carbon derived nanomaterials, as well as electrospun nanofibers have taken the lead in this area [5,8,10,11]. Regarding the use of nanoparticles, an extensive number of studies have described the properties of different nanomaterials such as magnetic nanoparticles, including iron oxide (Fe_3_O_4_ and γ-Fe_2_O_3_), alloy-based (CoPt_3_ and FePt), pure metal (Fe and Co), and spinel-type ferromagnets (MgFe_2_O_4_, MnFe_2_O_4_, and CoFe_2_O_4_) [12], or carbon derived nanoparticles, namely single and multiwall carbon nanotubes, graphene, graphene oxide, fullerene, etc. [4,13,14,15], as suitable carriers for enzymes of industrial interest. Similarly, applications for the use of these types of nanomaterials for the immobilization of prokaryotic and eukaryotic microorganisms have been investigated [11,16,17,18].

Among the different carbon-derived nanomaterials, graphene oxide has received a particular focus for biological applications because of its vast surface area, electroconductivity, superflexibility, and thermal stability, which makes this type of nanomaterial a suitable biological carrier [19,20]. Currently, it is possible to find in the market a portfolio of graphene oxide derivatives, expanding the availability of possible microbial and biomolecule immobilization materials for different applications. The use of distinct commercial graphene oxide nanoparticles can influence dramatically the biocatalyst loading, biochemical properties, and stability. For this reason, the selection of an optimal biocatalyst-carrier combination makes advisable a thorough screening of the available options [4]. Also, in regard to the suitability of graphene oxide derivatives as support for microbial immobilization, conflicting results relating biocompatibility and cytotoxicity induced by these nanomaterials have been reported in the literature [21], which could be in part due to their heterogeneity in functional groups composition, the presence of different amounts of trace elements, their size and morphology, etc. The fact that the materials used in most biocompatibility and toxicology studies are mostly homemade makes it challenging to achieve highly reproducible results. According to previous reports, graphene oxide nanoparticles have dose- and size-dependent toxicity toward different cell lines, such as human fibroblast, human hepatocellular carcinoma, human skin keratinocyte, etc. [22,23,24,25,26]. However, the amount of literature available focusing on the biocompatibility analysis of graphene with microbial cells is much scarcer.

In this research study we selected two graphene derivatives: monolayer graphene oxide (GO; supplied by Graphenea) and graphene oxide nanocolloids (GOC; supplied by Sigma-Merk), and both their toxicological potential against different unicellular organisms and their binding affinity toward different industrial enzymes was compared.

## 2. Results and Discussion

### 2.1. Characteristics of the Selected Commercial Graphene Oxide Derivatives

**Figure 1:**
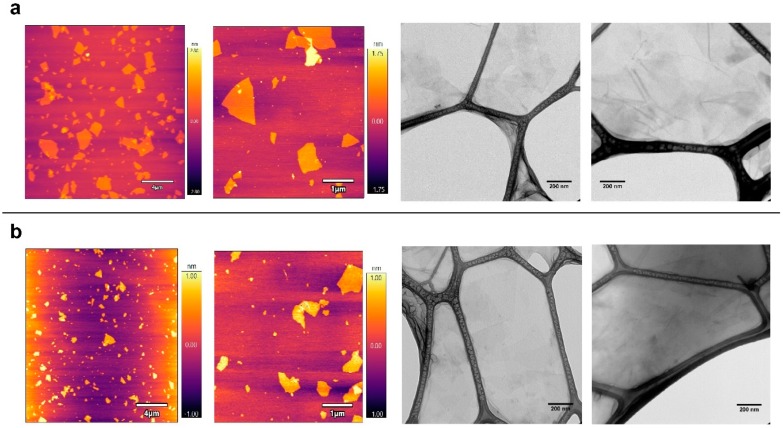
AFM and TEM analysis of graphene oxide (GO) (**a**) and graphene oxide nanocolloids (GOC) (**b**). Graphene suspensions with a final concentration of 20 mg L^−1^ were deposited by drop casting on a mica surface and carbon-coated copper grids respectively.

The physical-chemical properties of the graphene oxide derivatives selected for this study were recently determined [4]. Microscopy analyses using AFM and TEM instruments showed that GO and GOC flakes were mostly in monolayer state and had a different size, while the analysis of their composition revealed a high similarity between both nanomaterials. In the present study, the same commercial nanomaterials’ suspensions were selected, but a new batch of the GOC material was used (for more details see the Materials and Methods section). Therefore, we decided to perform a new microscopy and spectroscopy analysis to confirm the physico-chemical properties of the new GOC sample. Surprisingly, new AFM and TEM analyses revealed that the nanoparticles of the new GOC batch were morphologically very different to the older GOC batch (GOC_o_) (Supplementary Figure S1), showing instead a high similarity in morphology and size to that observed on the monolayer GO particles (Figure 1).

AFM topography imaging showed that both nanomaterial types have a wide lateral size distribution, ranging from the nanometric to the micrometric scale, while the flakes thickness is around 1–2 nm. Graphene oxide nanomaterials of similar characteristics have been reported to produce membrane-damaging activity in different unicellular systems [25,27,28].

**Figure 2:**
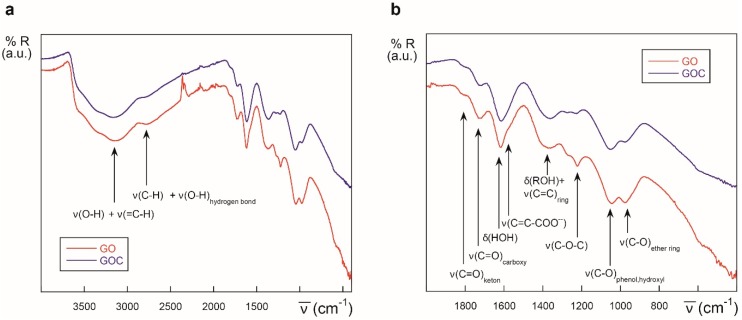
ATR-IR spectra of different graphene derivatives: GO (red) and GOC (blue), in the 4000–400 cm^−1^ (**a**) and 2000–400 cm^−1^ regions (**b**).

The FTIR spectra of GO and the new GOC batch was determined as well, and both nanomaterials showed to be very similar in their oxygen functional groups content (Figure 2). Following the tentative assignments given in the figure, the most significant difference found between GO and GOC was that the former showed a slightly greater content in ether/alcoxy groups than the latter, which could be related with the increase in the intensity of ν(C–O) stretching modes reported by other authors [29].

The results obtained indicate that the reproducibility in the production of commercial graphene oxide may still have relevant issues, making essential for the end user to confirm that the purchased product matches with the expected characteristics.

Since the presence of trace metal impurities in graphene derivatives, either contained in the graphite precursor or transferred by reactants used in the nanomaterial preparation, has been previously described, a trace element analysis of GO and GOC was done by inductively coupled plasma mass spectrometry (ICP-MS). As shown in Table 1, the presence of different metallic elements was observed in GO and GOC, although the concentration of most of them was found to be low. Nevertheless, significant differences in the concentration of some of the identified metals and metalloids were observed between both nanomaterials.

Overall, the concentration of metallic elements was higher in GOC than in GO. Both nanomaterials showed to have a high content of Mn (GO: 34.700 ppm; GOC: 62.405 ppm) and K (GO: 3.770; GOC: 2.628 ppm), which suggests they were obtained through the Hummer’s method, which is the most common oxidation method currently used for GO production and known to result in residual manganese accumulation because of the use of permanganate oxidant (KMnO_4_) [30]. Additionally, ICP-MS data suggested the possible presence of S in both nanomaterials, which can be present as well in graphene oxide prepared through the Hummer´s method, being its content significantly higher in GO. However, the obtained results in case of GOC were close to the background noise. For this reason, to get further insight into the possible presence of sulfur species and the differences in their content between GO and GOC, XPS analysis was performed. Again, the obtained results indicated that S species were higher in GO (relative atomic percentage: 0.6%) than in GOC, where a reliable quantitative value could not be determined. The presence of organosulfate groups in graphene oxide is described, and suggested to be responsible for part of the reactivity of this nanomaterial, such as in the immobilization of adsorbed species [31]. However, we could not get insights on the type of S species (e.g., organic or inorganic) present in GO or GOC.

### 2.2. Determination of Human Cancer Cell Line A549 Response to GO and GOC

**Figure 3:**
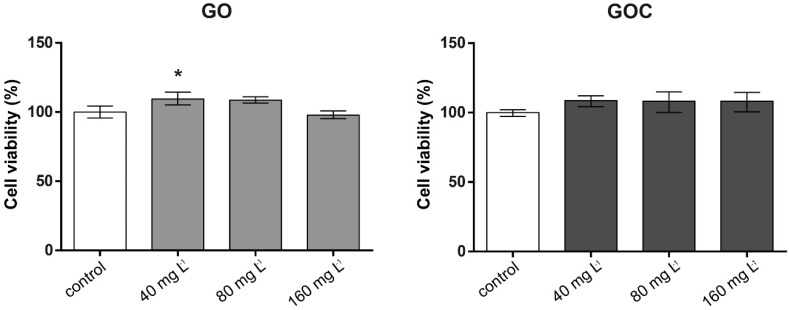
Viability of A549 cells (neutral red assay) treated with different concentrations of GO (**left**) and GOC (**right**). Results are expressed as % of control (untreated cells). Data represent the mean (±standard deviation, SD) of three independent replicates. Differences were established using a one-way ANOVA followed by Dunnett post hoc test to compare every mean with the control, and considered significant at *p* ≤ 0.05. * *p* ≤ 0.05.

The viability of the human cell line A549 after 24 h of exposure to 40, 80, and 160 mg L^−1^ of GO and GOC was analyzed using the neutral red uptake and MTT assays. The neutral red assay is based on the ability of healthy cells to incorporate and retain the neutral red dye in their lysosomes, which is an indicator of the cell’s capacity to maintain pH gradients through the production of ATP, and thus a viability indicator. In Figure 3, the results obtained for neutral red assay are presented. No negative effects on cell viability was observed in any of the concentrations tested for both nanomaterials, showing all the studied conditions (negative control and exposed cells) a similar percentage of viable cells.

**Figure 4:**
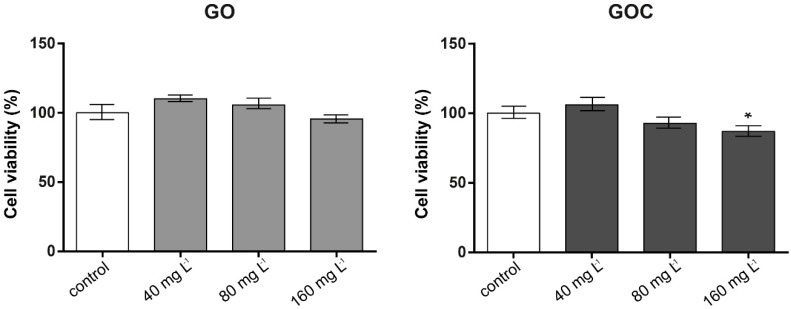
Viability of A549 cells (MTT assay) treated with different concentrations of GO (**left**) and GOC (**right**). Results are expressed as % of control (untreated cells). Data represent the mean (±standard deviation, SD) of three independent replicates. Differences were established using a one-way ANOVA followed by Dunnett post hoc test to compare every mean with the control, and considered significant at *p* ≤ 0.05. * *p* ≤ 0.05.

The MTT assay is based on the ability of viable cells with active metabolism to convert MTT into a purple colored formazan product that can be measured at OD 590 nm, being this color formation a useful marker to assess cells viability. The cytotoxicity studies conducted using this assay (Figure 4) revealed that cells exposed to GOC presented a slight decline in viability at the higher concentrations tested, being statistically significant in the case of cells exposed to 160 mg L^−1^, whereas in cells incubated with GO, no significant differences were found between controls and samples.

The toxicity of graphene oxide in human cell lines has been widely investigated in different studies. However, the results and conclusions reached by them are apparently inconsistent, as evidenced by some of the recent reviews [21,32]. Several factors, such as the size, the surface chemistry, or the levels of impurities, critically affect the physico-chemical properties of the nanoparticles and, subsequently, the interactions with cells, which lead to differences in their inherent cytotoxicity. Moreover, the toxicity of GO varies greatly depending on the cell line and cell type exposed [33]. In our experiments, only a slight statistically significant decrease in viability was detected in A549 cells treated with 160 mg L^−1^ of GOC (less than 15% of decrease) performing the MTT assay, whereas no negative effect was detected in the NR assay. It is also important to mention that in both assays a different number of cells per well were used, being six times lower in the MTT assay. Even in this case, where the nanoparticle/cell exposure ratio was higher, both GO and GOC demonstrated to be safe in terms of cell viability. These results are in concordance with the work of Chang et al. [34], which was performed using the same cell line. These authors described the good biocompatibility of GO, describing only a slight decrease in the viability after an exposure to high doses. In contrast, other authors observed a negative effect on the viability caused by these nanoparticles on A549 cells. Gies et al. described a size and dose dependent effect, showing a high decrease in the percentage of viable cells after 24 h of exposure to high concentrations of GO (100 and 200 mg L^−1^) [33]. Likewise, Reshma et al. showed a dose-dependent decrease in viability of cells treated with reduced GO (rGO) and PEGylated GO [35]. These authors observed a significant reduction from concentrations of, at least, 25 mg L^−1^. Mittal et al. analyzed the interaction between three graphene oxide derivatives with A549 cells [36], observing a significant reduction of viability over 48 h of exposure even at low concentrations, whereas Hu et al. described only a mild effect in cytotoxicity of A549 cells exposed during 24 h to GO and rGO, being significantly higher in the case of the latter [37]. This variability between the results obtained using the same cell line could be attributed to the factors explained above, such as the levels of impurities present in the nanoparticles, or even the oxidative method through which the nanoparticles were prepared, which influence their toxicological behavior [38].

**Figure 5:**
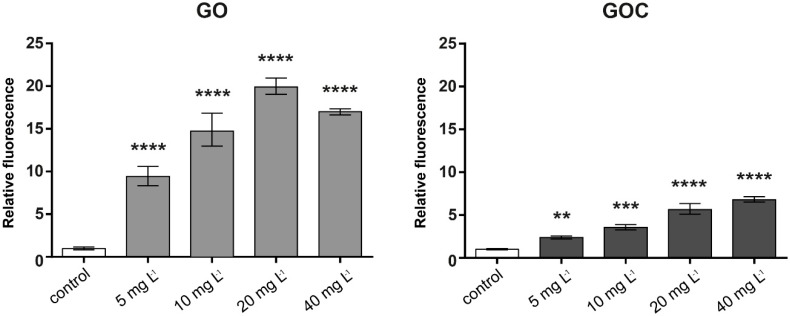
Reactive oxygen species (ROS) production of A549 cells treated with different concentrations of GO (**left**) and GOC (**right**). The reported values are expressed in arbitrary units and correspond to the averages of two biological replicates per culture condition. Data represent the mean of three replicates (±standard deviation, SD). Differences were established using a one-way ANOVA followed by Dunnett post hoc test to compare every mean with the control, and considered significant at *p* ≤ 0.05. ** *p* ≤ 0.01, *** *p* ≤ 0.001, **** *p* ≤ 0.0001.

In relation to the possible induction of oxidative stress by GO and GOC, the DCFH-DA assay was used to measure the reactive oxygen species (ROS) levels on the A549 cells after contact with different concentrations of the nanomaterials. Figure 5 shows that the ROS levels were significantly increased in A549 cells after 1 h of exposure to both nanoparticles, being this induction much higher in the case of the cells incubated with GO.

Our assays were performed using concentrations of both nanoparticle types up to 40 mg L^−1^. From that concentration, we have observed that in our experimental procedure the fluorescent response may be masked by both GO and GOC, leading to an underestimation of the ROS production. Either way, our results demonstrate that the low concentrations tested in our assays are enough to produce statistically significant levels of oxidative stress after 1 h of incubation, being this much higher in the case of GO. The induction of oxidative stress after interaction with graphene oxides and their derivatives have been reported in several works using different cell lines [39,40,41]. These nanomaterials can induce cellular damage through the formation of ROS by their interaction with cellular membranes. In the specific case of A549 cell line, several works have demonstrated their ability to induce ROS release. For example, Chang et al. found that GO exposure can induce oxidative stress at low concentrations [34]. Mittal et al. observed an overproduction of ROS in A549 cells in contact with GO and their derivatives, as well as in other human lung cells such as the BEAS-2B cell line [36]. In both studies, the times of exposure tested were longer than the times used in the present work. In any case, based on our results and in previous reports, it has been evidenced that an acute exposure of human cells to graphene oxide can induce high oxidative stress levels.

**Figure 6:**
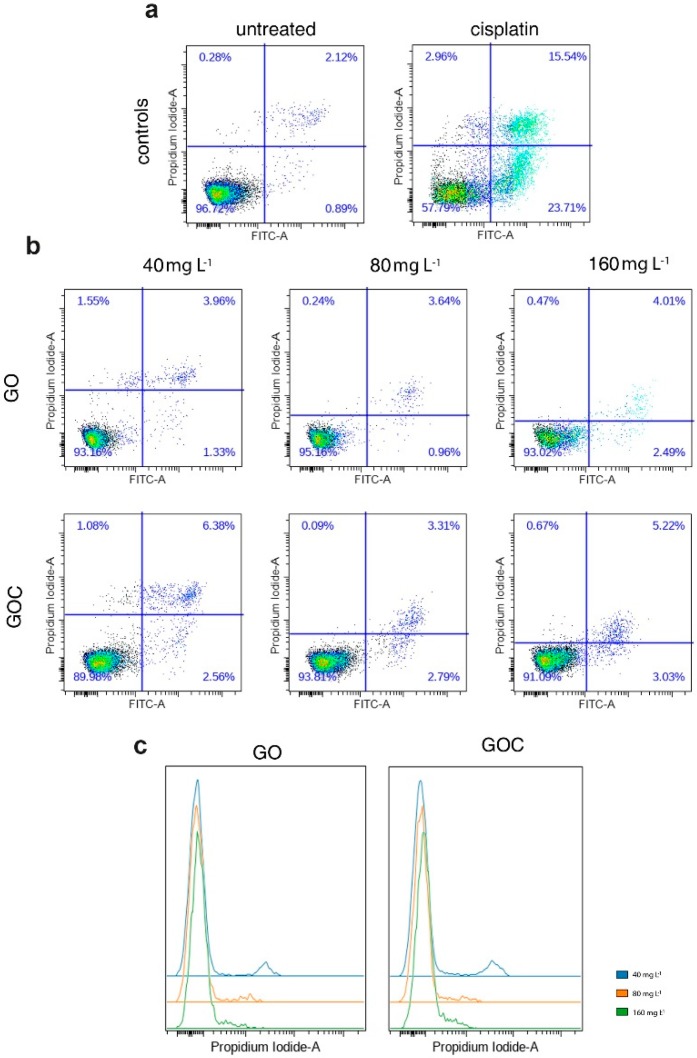
Flow cytometry analysis of apoptosis response of A549 cells treated with different concentrations of GO (top) and GOC (bottom) upon double staining with Annexin V-FITC and propidium iodide (PI). Results are displayed as density plots and expressed as percent (%) live (low left quadrants), apoptotic (low right quadrants), and necrotic (upper right quadrants) cells (**a**,**b**) of the total cell population excluding doublets. Histograms (**c**) show distribution of PI signal in cells treated with increased doses of GO and GOC.

High levels of ROS can cause damage to different biomolecules of the cell, such as proteins or nucleic acids, which can lead to activation of apoptosis. In order to assess whether the levels of ROS produced by A549 cells after being exposed to GO and GOC can induce an apoptotic response, we quantified the percentages of apoptotic and necrotic cells using flow cytometry, upon the addition of different nanoparticles concentrations for 24 h. The obtained results have shown that cells treated with different GO concentrations (Figure 6b; 40, 80, 160 mg L^−1^) showed a constant 93–95% of viable cells, similar to the untreated control sample (Figure 6a). In the case of GOC, we evidenced a stable 6–10% cell death, irrespective of the administered dose (Figure 6b). As a positive control for the assay, we used cisplatin (a common chemotherapeutic agent) which induced over 40% cell death (Figure 6a).

Interestingly, we found that the PI signal was decreasing in a dose-dependent manner in GO- and GOC-treated cells (Figure 6c). However, despite the signal to noise ratio diminution for the PI staining, this did not impede the quantification of the PI^+^ cell subpopulation. The PI signal decrease is probably caused by the quenching of the dye by the nanoparticles, as previously reported [42,43]. The quenching could be due to the energy transfer from the fluorophore to the metal [42] or in the case of graphenes, it could be due to the excitation of an exciton too [43]. Wu et al. found that the quenching efficiency of GO was still around 30% when the distance between dyes and GO was increased to more than 30 nm [44].

Several studies have described the impact of graphene-based materials on different types of programmed cell death, including apoptosis [45], in diverse cell lines, through distinct mechanisms such as caspase activation or DNA fragmentation [46,47]. For example, in the A549 cell line, the implication of graphene nanopores in the induction of early apoptosis was described and, at concentrations higher than 250 mg L^−1^, late apoptosis was observed too [48]. In addition, Adil et al. observed that apoptosis can be triggered by green synthesized nanocomposites of silver-decorated highly reduced graphene oxide [49], while Mbeh et al. described that high concentrations of graphene oxide nanoribbons (100 mg L^−1^) can also cause cell apoptosis [50]. However, other authors did not find any evidence of apoptosis induction in A549 cells after treatment with GO derivatives. For instance, Chang et al. observed that, independently of dose and size, GO did not induce any apoptosis or necrosis in A549 cells [34]. Moreover, Hu et al. described that apoptosis did not occur in A549 cells treated with GO nanosheets after a 24-h exposure with 20 and 85 mg L^−1^ [37]. Finally, Yang et al. found that the exposure to different graphene quantum dots, even at high concentration (200 mg L^−1^), did not result in apoptosis induction [51]. The results described in these latter works are in concordance with our observations, since, in spite of the fact that both GO and GOC produced oxidative stress in A549 cells, no significant increase in apoptosis was detected at concentrations up to 160 mg L^−1^.

### 2.3. Determination of Saccharomyces Cerevisiae Cells Response to GO and GOC

**Figure 7:**
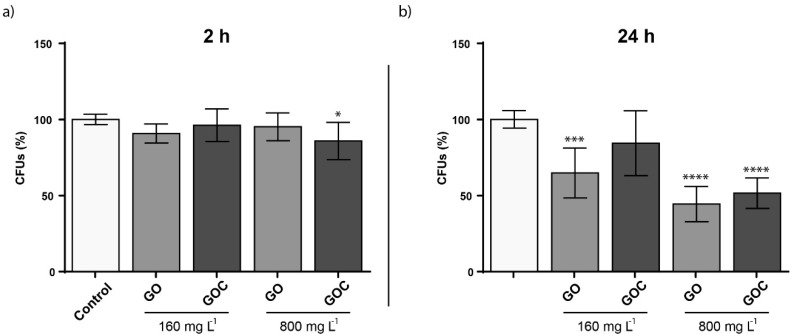
Colony forming units (CFUs) determination of *S. cerevisiae* cells exposed to 160 and 800 mg L^−1^ of GO and GOC during 2 h (**a**) and 24 h (**b**). The reported values are the averages of three biological replicates per culture condition. Differences were established using a one-way ANOVA followed by Dunnett post hoc test to compare every mean with the control, and considered significant at *p* ≤ 0.05. * *p* ≤ 0.05, *** *p* ≤ 0.001, **** *p* ≤ 0.0001.

The viability of *S. cerevisiae* cells exposed to two different GO and GOC concentrations (160 and 800 mg L^−1^) and exposure times (2 and 24 h) was assessed through colony forming units (CFU) determination. As displayed in Figure 7, no significant differences in viability were observed in the selected exposure conditions after 2 h of exposure, except for the condition where a high GOC concentration was used. However, after 24 h, viability issues could be observed after a longer exposure time. In case of GO, the nanomaterial reduced *S. cerevisiae* CFUs after an exposure of 24 h, provoking a viability loss of 36.5% when the material was present at the lower concentration and 49.7% when the material was present at the higher concentration. In contrast, GOC showed no significant influence on the yeast viability at 160 mg L^−1^, although the viability loss observed at the higher concentration was very similar for both nanomaterials. The effect on *S. cerevisiae* viability of non-commercial grade graphene oxide nanoparticles was also tested in a recent study, and the fungus mortality was found to be close to 20% in the presence of 600 mg L^−1^ [52]. Also, the toxicological potential of other carbon nanomaterials toward *S. cerevisiae* was reported, such as multi-walled carbon nanotubes (MWCNTs) or oxidized single-walled carbon nanotubes (O-SWCNTs), which induced significant yeast mortality at 400 mg L^−1^ (6.1%) and 188.2 mg L^−1^ (approximately 11%) respectively [53,54].

**Figure 8:**
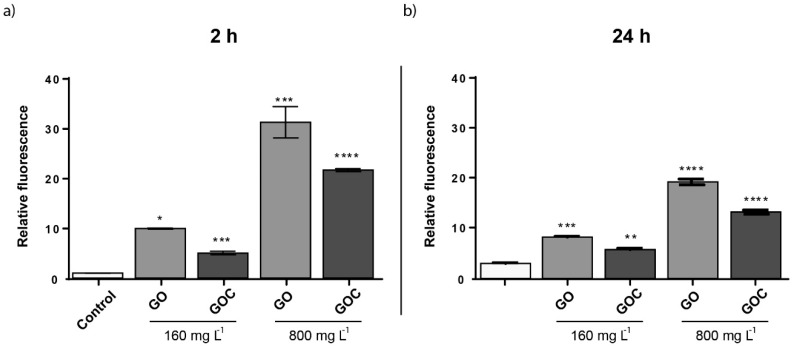
Oxidative stress (ROS) determination of *S. cerevisiae* cells exposed to 160 mg L^−1^ of GO and GOC during 2 h. The reported values are expressed in arbitrary units and correspond to the averages of two biological replicates per culture condition. Differences were established using a one-way ANOVA followed by Dunnett post hoc test to compare every mean with the control, and considered significant at *p* ≤ 0.05. * *p* ≤ 0.05, ** *p* ≤ 0.01, *** *p* ≤ 0.001, **** *p* ≤ 0.0001.

To evaluate whether GO and GOC were able to induce oxidative stress in *S. cerevisiae*, cells growing at exponential phase were exposed to 160 and 800 mg L^−1^ of the nanomaterials, for 24 h. As shown in the Figure 8, the oxidative stress levels were significantly increased in *S. cerevisiae* in the presence of both carbon nanoparticles. Carbon derived nanomaterials have shown previously to induce oxidative stress in yeast. Non-commercial grade GO and O-SWCNT, also induced ROS with a similar concentration to the one tested here, although the exposure time tested in both cases was 24 h instead of 2 h [52,54]. However, the oxidative stress provoked by MWCNT in yeast seem to be lower than that observed in the present study for GO and GOC or that previously observed for other carbon derived nanoparticles [53].

We also aimed to determine the possible genotoxic effect of the selected graphene oxide nanomaterials on *S. cerevisiae* using the comet assay protocol previously described [55]. However, because of the nanomaterials’ morphology, graphene oxide concentrations higher than 20 mg L^−1^ prevented the proper visualization and analysis of the cell nuclei under the fluorescence microscope, making the comet assay an unsuitable method for the determination of genotoxiciy in yeast with two dimensional nanoparticles of a big lateral size.

### 2.4. Determination of Vibrio Fischeri Bioluminescence Inhibition to GO and GOC

**Figure 9:**
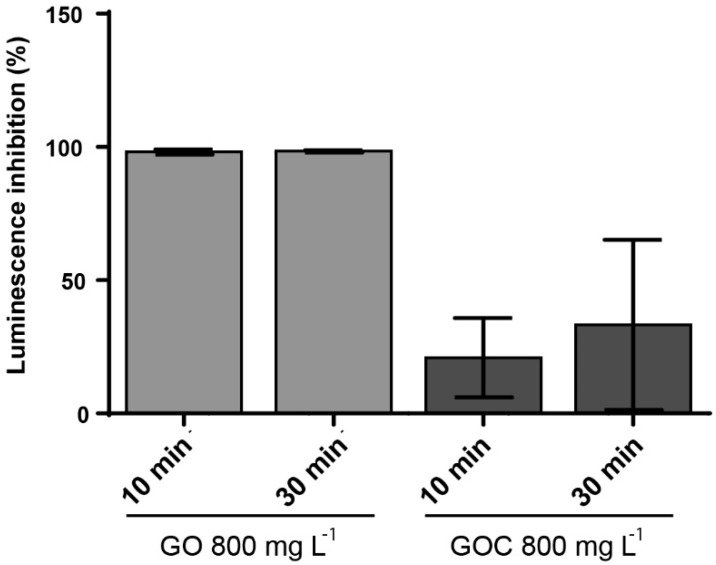
Luminescence inhibition assay of *V. fischeri* cells exposed to 800 mg L^−1^ of GO and GOC during 30 min. The reported values are the averages of four biological replicates per culture condition.

The marine bacteria *Vibrio fischeri* was also used to compare the toxicological potential of both graphene oxide suspensions. The *V. fischeri* luminescence assay is an environmental monitoring tool to determine the toxicity in sediments and leachates that may be a source of contamination in aquatic ecosystems. The ability of the nanomaterials to inhibit the microorganism luminescence was measured at two concentrations (160 and 800 mg L^−1^) and exposure times (10 and 30 min). When the lower concentration of GO and GOC was present in the media, we did not observe a *V. fischeri* significant luminescence inhibition. The bacteria luminescence decreased in the presence of a higher concentration of the nanomaterials, with significant difference between both nanomaterial types (Figure 9). In case of GO, the presence of 800 mg L^−1^ induced a 100% of luminescence inhibition, already after 10 min of exposure. In contrast, the same concentration of GOC showed a significantly lower luminescence inhibition capacity at both exposure times (*p* < 0.001 and *p* < 0.01 respectively).

Previous studies have evaluated the luminescence inhibition of *V. fischeri* promoted by nanomaterials, such as nano-metal oxides, nanoscale cationic polymers, silica nanoparticles, catechol-based submicron particles or functionalized reduced graphene oxide nanoparticles [56,57,58,59]. Interestingly, the toxicity of reduced graphene oxide functionalized with Fe_3_O_4_ [57], was similar to that observed for GOC in the present study.

### 2.5. Determination of GO and GOC Binding Efficiency on Different Microbial Enzymes

**Figure 10:**
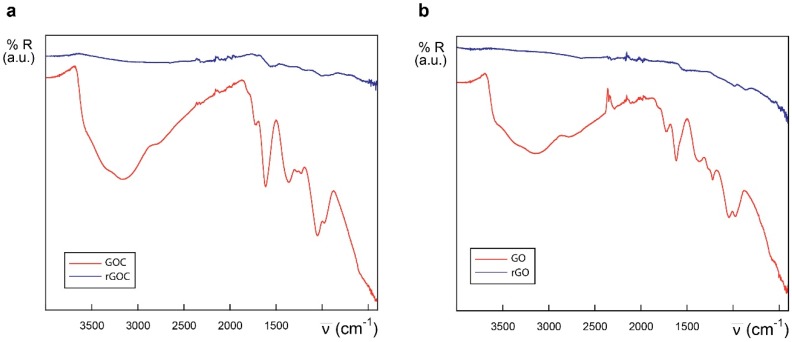
IR spectra of GOC and rGOC (**a**) and GO and rGO (**b**) in the 4000–400 cm^−1^ region.

Biotechnological and biomedical applications of graphene oxide rely on nanomaterial-biomolecule interactions. The protein binding capacity of nanomaterials determines possible biological applications and their toxicological potential too [60,61]. In case of commercial GO and GOC, both nanomaterial suspensions showed a high protein loading capacity and a good potential as enzyme immobilization supports [4]. However, their maximum protein binding capacity was not determined, and their polypeptide binding properties were determined using a single enzyme. Also, having into account that the protein binding efficiency of the new GOC lot (MKCD9594) was unknown, we decided to characterize the nanomaterial-enzyme binding efficiency of GO and GOC. In addition, to assess whether a variation on the GO and GOC oxidation state could further increase their enzyme loading capacity, the nanomaterials were partially reduced and their protein binding capacity was compared with that of the untreated nanomaterials. The partial reduction of GO and GOC was performed using a concentrated solution (50 mM) of the mild reductant mercaptoethylamine-HCl (further details are described in the Materials and Methods section). The reduction of the nanocarbon derivatives was confirmed by ATR-FTIR analysis (Figure 10). The spectrum of GOC exhibited drastic changes after the nanomaterials’ treatment with the mercaptoethylamine-HCl. Basically, the intensity of the absorptions sharply decreased, in good agreement with the reduction of the described functional groups. In the case of rGO, an analogous trend to that shown by the rGOC spectrum was observed.

The maximum enzyme loading capacity of chemically reduced GO (rGO) and GOC (rGOC) was analyzed and compared with that of the non-modified nanoparticles, using the bacterial enzymes α-l-rhamnosidase enzyme RhaB1, from *Lactobacillus plantarum*, and the β-d-glucosidase AbG, from *Agrobacterium* sp. (strain ATCC 21400), following the immobilization protocol described previously [4]. As displayed in Table 2, the binding capacity of GO and GOC was different for both enzymes and significantly higher than that observed in the reduced versions of the nanoparticles.

Although π–π stacking and hydrophobic effects are considered the predominant mechanisms of protein binding with graphene-based materials, and both phenomena should be more dominant after the reduction of graphene oxide, the reduced versions of GO and GOC did not improve the enzyme binding capacity of the untreated nanomaterials. Previous studies reporting the influence of graphene oxide reduction on protein binding capacity show controversial results [60,62,63,64]. As recently described by Qi and collaborators [64], changes on graphene-based nanomaterials’ surface properties affect as well their aggregation properties, which may become a crucial factor influencing their protein adsorption capacity. The obtained result also showed that the maximum loading capacity of GO and GOC was significantly higher for the α-rhamnosidase RhaB1. A similar result was observed when using the reduced versions. Different enzymes could exhibit different enzyme loadings and stabilities when bound to graphene oxide because of the differences in the charge status of their surface functional groups [65].

The obtained results using distinct unicellular models and biomolecules display significant changes in the toxicological potential of GO and GOC: the former had a higher ability to induce oxidative stress in human alveolar carcinoma epithelial cells A549, and the yeast *Saccharomyces cerevisiae*, while provoking a higher luminescence inhibition capacity on the bacteria *Vibrio fischeri* too. Also, both products behaved differently in their enzyme binding capacity. The lateral dimension, surface structure, functional groups, purity and protein corona, strongly influence the toxicity of graphene oxide in biological systems [66]. Since GO and GOC are distinct in terms of their apparent particle size distribution, elemental composition and in the presence of oxygen functional groups, identifying the most relevant factors determining the differences observed regarding their toxicological potential is difficult. Nevertheless, the present work contributes to have a better understanding on the biological impact and biotechnological potential of commercial grade graphene oxide.

## 3. Materials and Methods

### 3.1. Materials and Reagents

Most of the chemicals and reagents were purchased from Sigma-Aldrich (Merck KGaA, Darmstadt, Germany) and Acros Organics (Thermo Fisher Scientific Inc., Madrid, Spain). The graphene derivatives were obtained from different suppliers as well; graphene oxide nanocolloids (GOC; ref: 795534; old lot: MKBT5205V; new lot: MKCD9594) were purchased from Sigma-Aldrich, and monolayer graphene oxide (GO; C309/GORB014/D1) was purchased from Graphenea (San Sebastian, Spain). The α-l-rhamnosidase RhaB1 from *Lactobacillus plantarum* and the AbG β-d-glucosidase from *Agrobacterium* sp. (strain ATCC 21400) were obtained from Megazyme Ltd. (Biocon S.L., Barcelona, Spain).

### 3.2. ATR-FTIR Analysis

IR spectra were recorded on dry solid samples in the 4000–400 cm^−1^ region by a JASCO FT-IR 4200 spectrophotometer equipped with a Single Reflection ATR PRO ONE device. Each of the graphics is the result of overlapping 128 scans with a 4 cm^−1^ resolution.

### 3.3. ICP-MS

Samples (0.1 g) were subjected to a digestion process with 7 mL of HNO3 Suprapur (Merck KGaA, Darmstadt, Germany) (65% *v/v*) and 1 mL of H_2_O_2_ (30% *v/v*), while being subjected to the following thermal treatment: a temperature gradient from room temperature up to 80 °C in 4 min, followed by a second temperature gradient, from 80 to 120 °C in 4 min, and by a third temperature gradient, from 120 to 190 °C in 5 min. Then, temperature was kept constant at 190 °C for 30 min, and finally samples were cooled down for 1 h. The analysis of the digested samples was done with an Agilent 8900 ICP-QQQ instrument.

### 3.4. XPS Analysis

X-ray photoelectron spectroscopy (XPS) was done by the SGIker unit at the University of the Basque Country (UPV/EHU) using a SPECS system equipped with a Phoibos 150 on powders deposited into glass slides.

### 3.5. AFM and TEM Analysis

AFM and TEM analyses were performed at the Microscopy Unit from the University of Valladolid. Samples were deposited on Lacey Carbon Type-A, 300 mesh, copper grids, and visualized and photographed using a JEOL JEM-1011 HR TEM coupled with a Gatan Erlangshen ES1000W camera. For AMF analysis, samples were deposited on a mica surface from aqueous solutions by drop casting. Images were recorded in AC mode (tapping mode) with a CYPHER ES instrument from Asylum Research (Oxford Instruments, Abingdon, UK), using silicon cantilevers AC160TS-R3 with aluminum reflex coating (Olympus) and tip radius <10 nm. The analysis was done using a set point of 500, 72 mV, a drive amplitude of 791.16, a drive frequency of 268.639, and integral gain of 268.639. Data acquisition and control was done with IGOR Pro 6.2 (Asylum Research, Oxford Instruments, Abingdon, UK). Images analysis was done with ARgyle (Argyle Software Ltd., Bath, UK).

### 3.6. Assays in A549 Cells

The human alveolar carcinoma epithelial cell line A549 (ATCC, CCL-185) was utilized for toxicity evaluation. Cells were grown in DMEM medium (Dulbecco’s Modified Eagle Medium) supplemented with 10% fetal calf serum (FCS), 1% penicillin/streptomycin and grown in a humidified incubator at 37 °C in the presence of 5% CO_2_.

#### 3.6.1. Neutral Red Assay

A549 cells were seeded in 96 well plates at 3 × 10^4^ cells per well and treated with 40, 80, and 160 mg L^−1^ of the materials diluted in DMEM 1% FCS. After 24 h of exposure, cells were washed and incubated with 100 μL of the neutral red solution which was prepared as follows: neutral red stock (4 mg L^−1^) was diluted 1/100 in treatment media, and incubated in the dark for 24 h at 37 °C before use. At that time, the solution was centrifuged to remove debris from neutral red powder. After 2.5 h incubation, neutral red solution was discarded, cells were washed once with DPBS (Dulbecco’s phosphate-buffered saline), and subsequently fixed with formaldehyde 4%. Cells were washed again with DPBS and a dye release solution (50% ethanol 96°, 49% distilled H_2_O, and 1% acetic acid) was added to each well. After 10 min of gentle shaking, this solution was transferred to a new opaque 96-well plate, and fluorescence was measured with a microplate reader (BioTek Synergy HT, excitation wavelength, 530/25; emission wavelength 645/40). Results were expressed as percentage of control (absorbance of cells in absence of materials). Each assay included three independent replicates.

#### 3.6.2. MTT Assay

A549 cells were seeded in 96 well plates at 5 × 10^3^ cells per well and treated with 40, 80, and 160 mg L^−1^ of the materials diluted in DMEM 1% FCS. Cells incubated with medium alone were used as controls. Plates were then incubated for 24 h and, after exposure, cell culture medium with materials was discarded, wells were washed with DPBS, and a solution of MTT (3-(4,5-dimethylthiazol-2-yl)-2,5-diphenyltetrazolium bromide) (0.5 mg L^−1^) was added to each well and incubated for 3 h, followed by adding 100 µL DMSO to dissolve the MTT crystals. After 15 min of gentle shaking, the absorbance was measured with a microplate reader (BioTek Synergy HT, OD 590 nm). Results were expressed as percentage of control (absorbance of cells in absence of materials). Each assay included three independent replicates.

#### 3.6.3. ROS Determination in Human Cells

The quantitative measurement of intracellular reactive oxygen species (ROS) was investigated using 2,7-dichlorofluorescin diacetate (DCFH-DA). A549 cells were seeded in a 96 micro-well plate at 3 × 10^4^ cells per well and labelled with 50 μM DCFH-DA in Hanks’ Balanced Salt Solution (HBSS) for 30 min. After the incubation, cells were washed once with HBSS, and different concentrations of the materials diluted in HBSS were added to each well. Fluorescence was measured with a microplate reader (BioTek Synergy HT, excitation wavelength, 530/25; emission wavelength 645/40) after 1 h of incubation.

#### 3.6.4. Apoptosis Assay

Flow cytometry was used for the quantitative assessment of apoptosis. A549 cells were seeded in 24 well plates at 10 × 10^4^ cells per well and treated with 40, 80, and 160 mg L^−1^ of the materials diluted in DMEM 1%FCS. Cells incubated with medium alone were used as negative controls while cells treated with 50 µM cisplatin served as positive control for the staining. After 24 h of incubation, cells in suspension were harvested and collected together with the monolayers detached using trypsin-EDTA solution (Invitrogen), for each sample. After centrifugation, cells were resuspended in buffer and stained using a dead cell apoptosis kit with Annexin V-FITC and propidium iodide (Molecular Probes) according with manufacturer’s protocol. Samples were filtered through 70-µm nylon meshes (Miltenyi Biotec) and acquired on a BD FACSVerse analyzer controlled by FACSuite software (BD Biosciences, Franklin Lakes, United States). Analysis was performed on the Cytobank platform (https:\\community.cytobank.org). Single stained controls, using Triton-X-100 permeabilized (0.2% in PBS, 10 min) and untreated cells, respectively were generated for compensation purposes and gating thresholding. Results are depicted as color density plots and histograms.

### 3.7. Assays in Saccharomyces Cerevisiae

The *S. cerevisiae* BY4741 strain was grown and maintained in standard liquid YPD medium (1% yeast extract, 1% yeast bacto-peptone, 2% glucose). Cell cultures in liquid media were done on a rotary shaker at 185 rpm at 30 °C.

#### 3.7.1. Colony Forming Units Determination

Yeast cells in exponential growth phase (OD600 = 1) were exposed to GO and GOC at 160 and 800 mg L^−1^ in 1 mL cultures performed in 24-well plates. Samples were obtained after 2 and 24 h of cells exposure. To determine yeast colony forming units after the two exposure times, cells were inoculated on solid YPD medium (6% agar) and incubated at 30 °C.

#### 3.7.2. ROS Determination in *S. cerevisiae*

Intracellular levels of reactive oxygen species were determined using the reagent CM-H2DCFDA following a protocol similar to that reported by James et al. (2015) [67]. *S. cerevisiae* cells growing in exponential phase were pelleted, washed, and incubated with CM-H2DCFDA (7 μM) in DPBS for 60 min at 30 °C and 185 rpm. Afterwards, yeast cells were washed again, resuspended in YPD and subsequently exposed to the graphene oxide nanomaterials (160 mg L^−1^) for 2 h. Then, yeast cells were washed two times with DPBS, incubated 2 min in a solution containing AcLi 2M, and subsequently washed and incubated again for 2 min in a solution containing SDS (0.01%) and chloroform (0.4%). Finally, cells were pelleted and the supernatant was transferred to a black opaque 96-micro-well plate, where the fluorescence was measured (excitation = 485; emission = 528) using a microplate reader (Synergy-HT, BioTek).

### 3.8. Vibrio Fischeri Luminescence Inhibition Assay

*V. fischeri* NRRL B-11177 cells were inoculated in 5 mL of Marine Broth 2216 and grown at 15 °C for 48 h. The bacterial suspension was pelleted, resuspended in 5 mL of NaCl 2% (*w/v*) at 15 °C and maintained at 10 °C for 30 min. The exposure experiment was started by pipetting 10 µL of the bacterial suspension in black opaque microplate wells containing 90 µL of GO and GOC (160 and 800 mg L^−1^) in a water suspension containing NaCl 2% (*w/v*). The 96-well plate was incubated in a Thermomixer at 800 rpm and 15 °C, and *V. fischeri* luminescence was measured for 30 min using a microplate reader (Synergy-HT, BioTek). The luminescence inhibition (using as reference the negative control condition) was calculated using the values obtained at 10 (M10) and 30 (M30) min using the following formula, adapted from Jarque et al. (2016) [68], where CF is a correction factor (the Mt/peak ratio in negative controls) reflecting natural attenuation of bacterial luminescence after 30 min of incubation in non-exposed conditions:

$$INH\%=100-\frac{Mt}{CF\times peak}\times 100$$

### 3.9. Preparation of rGO and rGOC

The mild reductant mercaptoethylamine-HCl was used to reduce commercial GO and GOC nanoparticles. Water suspensions of GO and GOC (1000 mg L^−1^) containing 50 mM of the reducing agent concentrated were incubated overnight at 4 °C. Afterwards, rGO and rGOC were pelleted, using a Thermo ST 16R Sorvall centrifuge (5000 rpm; acceleration: 9, deceleration: 9), and subsequently washed with a sodium phosphate buffer (12.5 mM; pH 6.5) solution, three times. Finally, the reduced nanomaterials water suspensions were kept at a final concentration of 1000 mg L^−1^ in sodium phosphate buffer (12.5 mM; pH 6.5), and stored at 4 °C.

## 4. Conclusions

The results obtained in the present study show the potential of different commercial graphene oxide nanomaterials to interact with distinct unicellular systems and biomolecules, pointing out the variability that can be found in terms of toxicological potential and binding affinity depending on the target organism or protein, and the selected nanomaterial. GO showed a higher capacity than GOC to induce oxidative stress in both *S. cerevisiae* and human cells. In the same line, GO showed a significantly higher *V. fischeri* luminescence inhibition too. Also, differences in the binding capacity of both nanomaterials were observed, being their maximum loading capacity different as well, in function of the enzyme tested. Therefore, the presented results clearly indicate the usefulness of this type of studies in order to determine the actual toxicological and biochemical potential for specific commercial graphene oxide products.

## Figures and Tables

**Table 1:** Inductively coupled plasma mass spectrometry (ICP-MS) analysis of GO and GOC. Values below the detection limit of the ICP-MS procedure are also shown.

	GO (ppm)	GOC (ppm)
Al	0.160 ± 0.113	1.445 ± 0.106
B	<0.004	1.600 ± 0.255
Ba	0.006 ± 0.008	0.214 ± 0.006
Ca	0.063 ± 0.088	0.835 ± 0.035
Cu	0.052 ± 0.039	0.581 ± 0.030
Fe	0.379 ± 0.067	1.899 ± 0.033
Ga	0.004 ± 0.006	0.047 ± 0.000
K	3.770 ± 0.184	2.628 ± 0.252
Mg	0.350 ± 0.028	2.000 ± 0.113
Mn	34.700 ± 0.156	62.405 ± 0.233
Mo	0.029 ± 0.002	0.017 ± 0.001
Na	1.240 ± 0.509	4.810 ± 0.057
Ni	0.027 ± 0.020	0.027 ± 0.007
Pb	0.054 ± 0.023	0.152 ± 0.009
S	43.200 ± 2.786	5.084 ± 2.752
Sn	0.003 ± 0.003	0.034 ± 0.001
Sr	0.008 ± 0.001	0.034 ± 0.001
V	<0.0001	0.006 ± 0.001
W	0.004 ± 0.001	0.006 ± 0.001
Zn	0.068 ± 0.061	1.069 ± 0.740

**Table 2:** Maximum binding capacity (%) of GO, GOC, rGO, and rGOC using different carbohydrate active enzymes.

Carbon Nanomaterial	RhaB1 Binding (mg mg^−1^)	AgB Binding (mg mg^−1^)
GO	4.88 ± 0.17	1.65 ± 0.04
GOC	5.90 ± 0.11	1.22 ± 0.14
rGO	1.98 ± 0.11	1.00 ± 0.03
rGOC	1.99 ± 0.23	0.70 ± 0.08

## Supplementary Materials

Supplementary Materials can be found at https://www.mdpi.com/1422-0067/21/1/205/s1.

## Abbreviations

GO	Monolayer graphene oxide
GOC	Graphene oxide nanocolloids
ROS	Reactive oxygen species
AFM	Atomic force microscopy
TEM	Transmission electron microscopy
FTIR	Fourier-transform infrared spectroscopy
ICP-MS	Inductively coupled plasma mass spectrometry
ppm	parts-per-million
ATP	Adenosine triphosphate
SD	Standard deviation
PI	Propidium iodide
MWCNs	Multiwalled carbon nanotubes
O-SWCNTs	Oxidized single-walled carbon nanotubes
rGO	Reduced monolayer graphene oxide
rGOC	Reduced graphene oxide nanocolloids
DMSO	Dimethyl sulfoxide
