# Supplementary material for: Interaction Analysis of Commercial Graphene Oxide Nanoparticles with Unicellular Systems and Biomolecules
Source: Int J Mol Sci. 2019 Dec 27;21(1):205. doi: 10.3390/ijms21010205 (PMC6982217; doi:10.3390/ijms21010205)
Supplement: Supplementary file 1 [file ijms-21-00205-s001.docx]

**
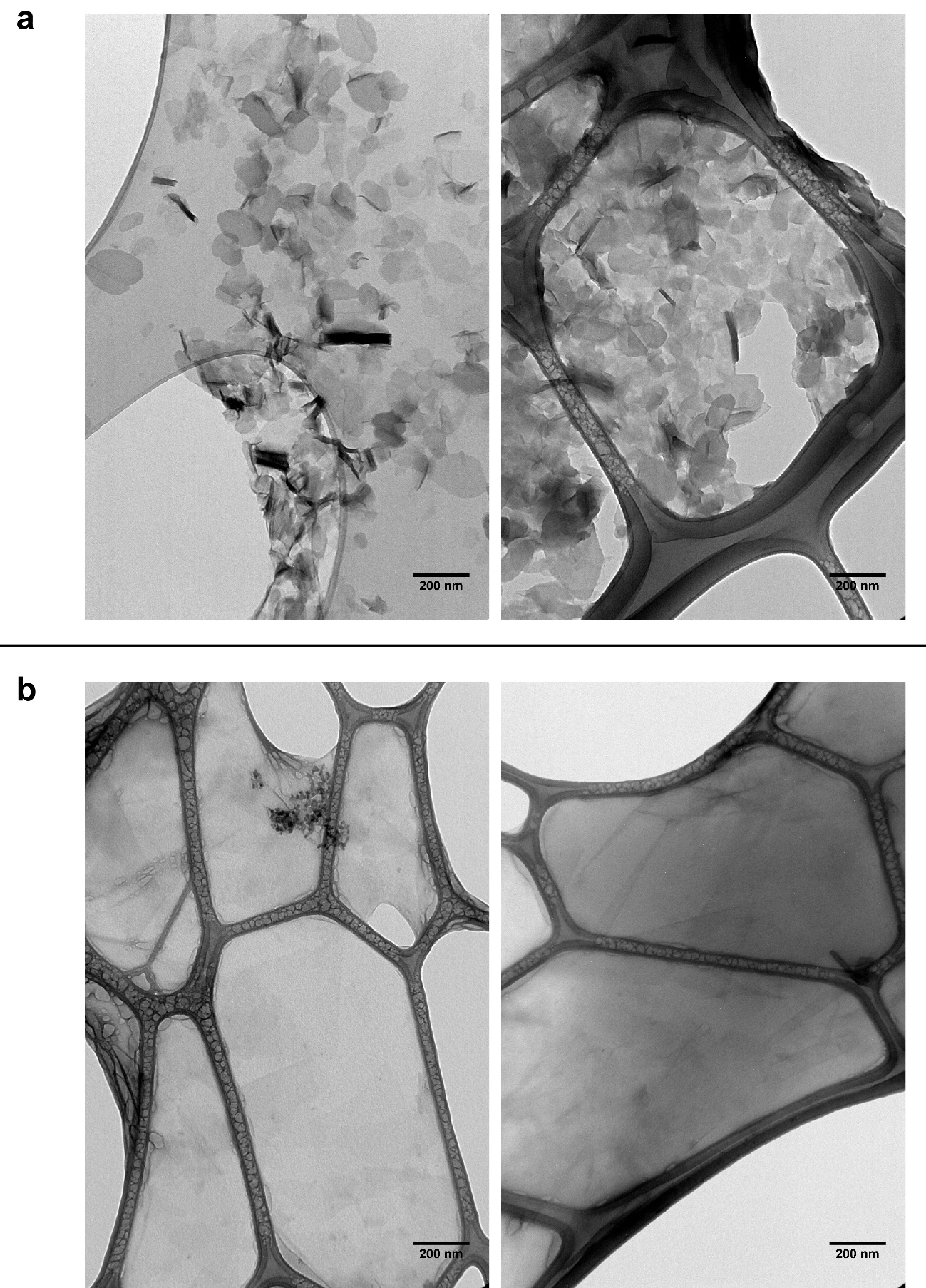
**

**Figure S1.** TEM analysis of two different lots of commercial graphene oxide nanocolloids (ref: 795534): (a) GOC_o_ (lot: MKBT5205V) and (b) GOC (lot: MKCD9594).
